# High myopia control is comparable between multifocal rigid gas-permeable lenses and spectacles

**DOI:** 10.3389/fmed.2023.1207328

**Published:** 2023-08-10

**Authors:** Li-hua Yu, Ran Zhuo, Guan-xing Song, Meng Lin, Wan-qing Jin

**Affiliations:** ^1^National Clinical Research Center for Ocular Diseases, Eye Hospital, Wenzhou Medical University, Wenzhou, China; ^2^School of Ophthalmology & Optometry, Wenzhou Medical University, Wenzhou, Zhejiang, China; ^3^The First People’s Hospital of Aksu District in Xinjiang, Aksu, China

**Keywords:** axial length, high myopia, multifocal rigid gas-permeable lens, myopia control, lens design

## Abstract

**Purpose:**

Ocular pathology may be reduced by slowing myopia progression. The purpose of this study was to evaluate the potential of a novel custom-designed rigid gas permeable (RGP) contact lens to control high myopia by comparing the efficacy of multifocal RGP lenses and single-vision spectacles for high myopia control.

**Methods:**

The medical records of children fitted with spectacles or multifocal rigid gas-permeable lenses between January 2018 and May 2020 were retrospectively reviewed. Children (5–17 years) with non-cycloplegic spherical equivalent refraction of ≤ −6.00 D or spherical equivalent refraction > − 6.00 D with baseline axial length ≥ 26.5 mm, and astigmatism of ≥ −2.00 D were included. Axial length and refraction were measured at baseline, before fitting the participants with multifocal rigid gas-permeable lenses or spectacles, and at 1- and 2-year follow-up visits. Changes in axial length were compared between the groups.

**Results:**

Among the 77 children with 1-year follow-up data, the mean axial elongation was 0.20 ± 0.17 mm and 0.21 ± 0.14 mm in the multifocal rigid gas-permeable and control groups, respectively, without significant differences between groups (*F* = 0.004, *p* = 0.835). Among the 41 patients who completed 2 years of follow-up, the mean axial elongation values in the multifocal rigid gas-permeable and control groups were 0.21 ± 0.15 mm and 0.24 ± 0.13 mm, respectively, at the 1-year follow-up, and 0.37 ± 0.27 mm and 0.43 ± 0.23 mm, respectively, at the 2-year follow-up, without significant between-group differences at either time point (*p* = 0.224).

**Conclusion:**

Axial length increased at a similar rate in both the control (spectacles) and multifocal rigid gas-permeable lens groups, suggesting that multifocal rigid gas-permeable lenses have no significant impact on controlling high myopia progression compared with spectacles.

## 1. Introduction

Myopia, a refractive error that is highly prevalent globally, is increasingly prevalent in young adults and adolescents. It is associated with increased risks for several other ocular conditions, including cataracts (1, 2), glaucoma (3), retinal detachment, and maculopathy (4). Thus, myopia represents a serious public health burden, and controlling its progression has garnered considerable research attention (5, 6). Accordingly, several strategies have been formulated to impede or halt the development of myopia in younger adults. These methods generally fall into two categories: topical pharmaceutical agents (7–9) and optical treatments, including bifocal and multifocal spectacles (10–12) and contact lenses with power profiles that produce peripheral myopic defocus (13–15). However, there are limitations with respect to the safety, efficacy, affordability, and/or application of these strategies. In recent years, it have been noted that novel designed Spectacle lenses are available to retard myopia progression, such as Defocus Incorporated Multiple Segments (DIMS) lens and highly aspheric lenslets (HAL) lens, its myopia control effects have been observed in both low and moderate myopes (12, 16, 17). Currently, novel designed lenses have been increasingly used in the clinic for it’s good acceptance, adaptability, safety and relatively low cost among children and parents. However, several studies have showed that the DIMS lenses myopia control efficiency was diminished with the increasing baseline spherical equivalent (SE) among children (18, 19). Orthokeratology lenses are expensive and require close attention to safety and maintenance owing to the increased risk of infection when wearing contact lenses overnight. There are no available products for individuals with very high refractive errors. Multifocal soft contact lenses (MFSCLs) are also too expensive for many families, and parents must demonstrate appropriate contact lens handling skills for safe and successful lens wear. Additionally, individuals with high refractive errors (such as <−10.00 D) cannot obtain suitable products, and those with astigmatism greater than 1.50 D may not achieve satisfactory visual acuity (20). Several studies have demonstrated that the use of 0.01% atropine eye drops alone did not have a noticeable effect on controlling axial growth in children and adolescents aged 6–12 years (21–23). However, if the concentration of atropine increases, the effect of controlling myopia may be enhanced while the side effects, including photophobia, near vision blurring, and rebound effect after stopping the use of atropine eye drops will be become more pronounced (24). In mainland China and other regions, commercial low-concentration atropine eye drops products are still not easily accessible (8, 25). Therefore, prevention of myopia and its progression may require different approaches depending on the individual circumstances and geographic regions (26).

In recent years, several studies have reported that peripheral defocus soft contact lens designs can limit axial elongation and inhibit myopia progression in children, compared with controls (13, 27). In addition, orthokeratology lenses were found to delay low-to-moderate myopia progression (28–30). Nonetheless, to date, only moderate-concentration atropine eye drops and partial reduction orthokeratology have been reported to be effective in controlling high myopia progression (31–33).

Studies on animals have demonstrated that optically induced changes to the effective refractive status of the eye can regulate eye growth and impact the development of refractive errors (34). Orthokeratology and peripheral defocus soft contact lens mechanisms are based on the theory that hyperopic defocus on the peripheral retina induces excessive eye growth and myopia, while myopic defocus reduces this growth (13). Therefore, induction of peripheral myopic defocus has consequently become the mainstay of many current myopia control strategies, and daily wear of a newly developed multifocal rigid gas-permeable (mRGP) lens, designed by Eagle Vision Technology, Co., was also based on this theory.

This study aimed to investigate the efficacy of this novel custom-designed mRGP lens compared to single-vision spectacles for controlling axial length growth and reducing myopia progression in children and adolescents with high myopia.

## 2. Materials and methods

### 2.1. Study design and population

This was a retrospective analysis of the medical records of children and adolescents with high myopia who were consecutively fitted with mRGP lenses or single-vision spectacles between January 2018 and May 2020 and were followed up for at least 1 year. The control group was selected from the same time frame and matched to the RGP group for age, refractive errors, and baseline axial length. Patients were categorized into mRGP and control groups, and data were compared between the groups at the 1- and 2-year follow-up stages.

Patients were selected based on the following inclusion criteria (Table 1): (1) high myopia with spherical equivalent refraction ≤ −6.00 D (non-cycloplegic sub-refraction) or baseline axial length ≥ 26.5 mm (35), which includes both axial myopia and refractive myopia; (2) corrected acuity ≥6/6 after wearing mRGP lenses; and (3) normal anterior segment, fundus, and other ocular structures at initial examination. Factors that could affect the study results were included in the exclusion criteria: (1) patients in the mRGP group who discontinued wearing lenses for more than 1 month during the follow-up period; (2) patients who used low-concentration atropine eye drops or wore defocus-based spectacle lenses or multifocal contact lenses during the follow-up period. Age, sex, and spherical components were considered to limit the systematic bias when selecting and matching the study participants.

**Table 1:** Inclusion criteria.

	Condition
Age	5–17 years (inclusive) at baseline
Ocular health	Apart from myopia, no ocular diseases such as obvious tropia, retinopathy, prematurity, or fundus changes. No organic changes except for leopard-like retinal patterns	Intraocular pressure < 21 mmHg Corrected visual acuity ≥6/6
Refractive error	High myopia with a spherical component ≤ − 6.00 D or baseline axial length ≥ 26.50 mm with a spherical component > −12.00 D	Astigmatism ≥ −2.00 DC
History	None for orthokeratology or other optic and drug treatments (e.g., low-concentration atropine eye drops)
Other	No medications affecting refractive development	Complete and recorded 1- or 2-year follow-up examinations	No strabismus from cover-uncover tests; with or without refractive correction

DC, diopter of cylinder.

The study adhered to the tenets of the Declaration of Helsinki and was approved by our local ethics committee (approval number KYK[2022]164).

### 2.2. Materials

**Figure 1:**
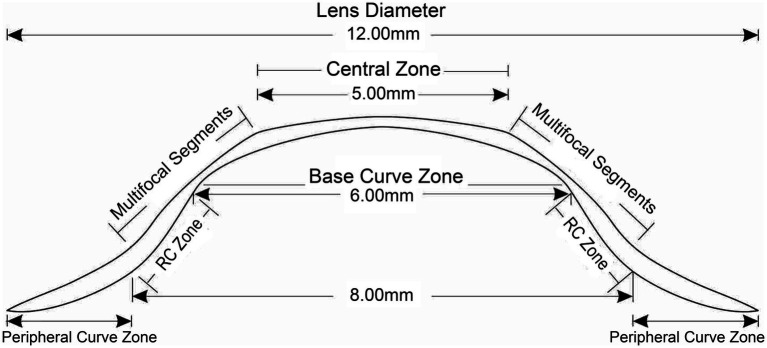
Multifocal rigid gas-permeable lens design. RC zone, reverse-curve zone; PC, peripheral curve.

**Figure 2:**
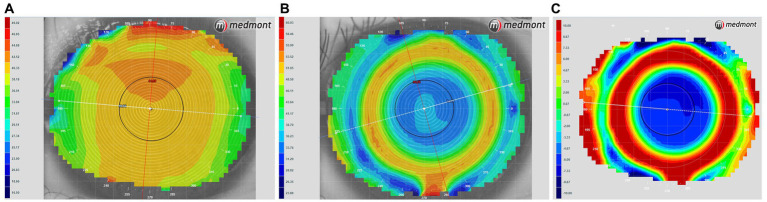
Corneal topography tangential eye maps obtained **(A)** before fitting and **(B)** while wearing multifocal rigid gas-permeable lenses. **(C)** A power difference map showing alterations in corneal power after lens fitting; warmer colors indicate higher corneal power.

Multifocal Dyna EO RGP contact lenses (Eagle Vision Technology. Co, Taipei, China) were used in this study. The lenses are made of Boston EO (enflufocon B comprising an aliphatic fluoroitaconate siloxanyl methacrylate co-polymer with an ultraviolet absorber) with an oxygen permeability co-efficient of 82 × 10^−11^ (cm^2^ × ml O_2_)/(s × ml × mmHg). This day-wearing lens has design features similar to those of modern reverse-geometry orthokeratology lenses, which reduce peripheral hyperopic defocus (Figure 1). However, the back base- curve was designed to be parallel to the corneal shape and not to flatten the central cornea, with the addition of a plus lens to the mid-peripheral curve on the anterior surface, in order to induce a shift in myopic defocus on the peripheral retina. The mRGP lens contains a central zone that corrects refractive errors and concentric treatment zones that add 9.00 D positive lenses and induce peripheral myopic retinal defocus. The corneal topography changes before fitting and wearing the mRGP lenses are shown in Figure 2.

The mRGP lens design characteristics are as follows. The central optical zone diameter is 5 mm. The anterior curvature begins to reverse 2.5 mm from the lens center, and the reversal degree becomes larger; the peripheral curve radius of the anterior surface is at 85–92% of the central curve radius at 3.5 mm from the lens center. The mid-peripheral refractive power of lens is increased by +9.00 D compared to the central optical zone (For example, when the refractive power at the center of the lens is −5.00D, the maximum refractive power at the mid-peripheral zone is +4.00D). The central posterior surface within 6 mm is spherical, and the posterior curve begins to reverse 3–4 mm from the lens center (the reverse-curve zone). Starting at 4–5 mm from the center, the posterior surface changes to a conical surface, as defined by the peripheral curve. The lens specifications are listed in Table 2.

**Table 2:** Multifocal rigid gas-permeable lens specifications.

	Multifocal RGP lenses with peripheral defocus
Manufacturer	EagleVison Technology, Co.
Material	Boston EO material
Water content	<1%
Base curve (mm)	5.00 to 11.00
Wetting angle	49°
Oxygen permeability (cm^2^ × ml O_2_)/(s × ml × mmHg)	82 × 10^−11^
Total diameter (mm)	7.00 to 12.00
Power (D)	−20.00 to 0
Add (D)	+9.00

RGP, rigid gas-permeable; D, diopter.

### 2.3. Study procedures

In all patients, the mRGP lenses were fitted by experienced practitioners. The initial fit was determined according to the diagnostic evaluation. Appropriate trial lenses were chosen according to corneal curvature and visible horizontal iris diameter (HVID). The fitting assessment included static and dynamic fit after an adaptation period of approximately 20 min. Static fit was assessed by fluorescence; a satisfactory fit showed very slight central clearance with sufficient edge width and clearance. Next, optimal dynamic fitting was done: the lens was well-centered (less than 0.5 mm from the center) with 0.5–1.5 mm smooth vertical movement with blinking. When the patients achieved ideal fitting with the trial mRGP lenses, subjective refraction using MPMVA was performed by adding spectacle lenses to the patients with trial lenses; the final mRGP prescription was determined by the power of the trial lens and added spectacle lenses. After the lenses were dispensed, the patients were advised to wear them daily and were provided with clear instructions regarding the wearing and maintenance of the lenses. The patients were also instructed to wear mRGP lenses or spectacles for at least 8 h per day.

Follow-up examinations were performed at 1 week and 1 month after dispensing the lens, and every 3 months thereafter. At each visit, a Snellen chart was used to assess visual acuity, and slit-lamp examinations were conducted to assess lens integrity and corneal health. Medmont E300 instrumentation (Medmont program V6; Medmont International Pty Ltd., Melbourne, Victoria, Australia) was used to monitor changes in corneal morphology. The mRGP lenses were generally replaced at 1–1.5 years.

**Figure 3:**
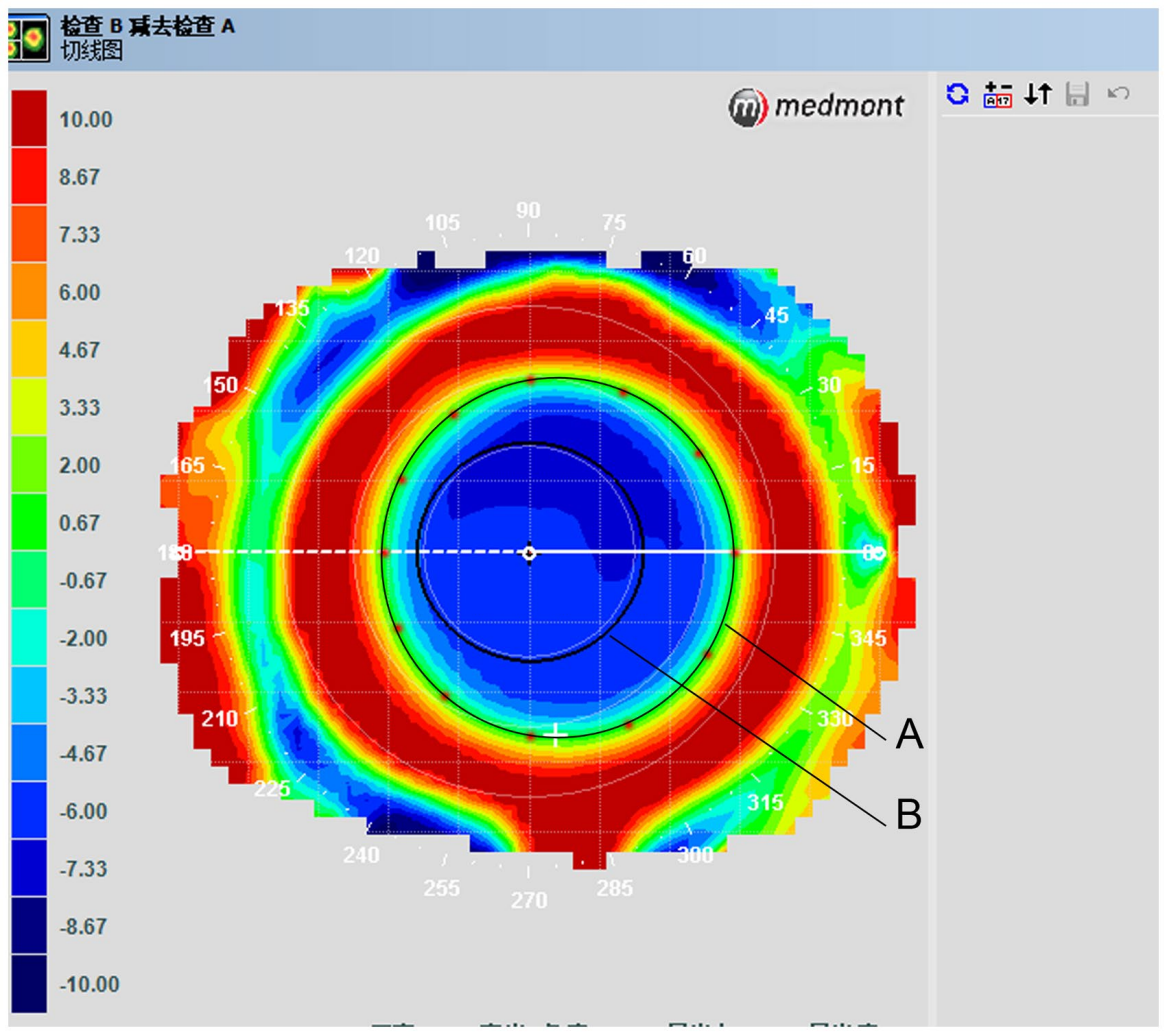
Tangential difference topographic map showing average treatment zone diameters. **(A)** Treatment zone border curve. Treatment zone borders, which were defined as transition points from negative to positive values, were taken from tangential difference maps. **(B)** Pupil diameter.

Treatment zones/locations were assessed as follows. Corneal topography was examined at baseline and 1 month after the lenses were prescribed. This step was performed with the lenses *in situ*. Treatment zone borders, defined as transition points from negative to positive values, were manually extracted from the tangential difference maps. The distances from these points along the meridians of 0, 30, 60, 90, 120, 150, 180, 210, 240, 270, 300, and 330 degrees to the corneal center were averaged, and twice the value was deemed the central treatment zone (Figure 3). Pupil size was determined using topographic data under ambient mesopic illumination, but photopic conditions were used due to the intrinsic light levels of the topographer. Pupil size was determined using the average horizontal and vertical pupil diameters.

Axial length was measured and recorded in all patients at the 1- and 2-year visits. Measurements were performed using non-contact optic biometric instrumentation (IOLMaster; Carl Zeiss Meditec AG, Jena, Germany). For each test, five successive measurements were performed within 0.05 mm of one another, and the mean representative values were used. In addition, it was ensured that the minimum signal-to-noise ratio for each measurement was >2.

### 2.4. Sample size calculation

For adolescents and children, an increase in axial elongation of approximately 0.1 mm/yr. is generally considered to be associated with normal eye growth, while an increase of 0.2–0.3 mm/yr. is associated with increasing myopia (20); thus, in this study, it was assumed that in comparison to the single vision spectacle group (control group), a slowing of 0.2 mm/yr. (approximately 0.50 D) in axial elongation in the mRGP group would indicate sufficient control of myopia progression. Groups were established to generate 90% power in detecting a minimum difference of 0.20 mm in axial elongation at the 1-year follow-up at 5% statistical significance using a standard deviation of 0.27 mm, based on a previous study by Zhu (36). The minimum sample size required was 32 patients for each group.

### 2.5. Statistical analysis

Axial length changes were compared between the mRGP and control groups. As data were normally distributed (Kolmogorov–Smirnov test; *p* > 0.05), parametric tests were used. Unpaired t- and chi-square tests were used to compare baseline data and differences in male/female ratios between the groups. To assess myopia progression, univariate covariance and repeated-measures one-way analysis of variance were used to compare axial length changes within the groups over time and axial elongation values between the groups. The mean and standard deviation values for continuous variables and all other variables were calculated using IBM SPSS Statistics (ver. 23, IBM Corp., Armonk, NY, USA). Two-sided value of *p* <0.05 were considered statistically significant.

## 3. Results

A total of 77 children and adolescents (150 eyes) were included: 38 in the mRGP group and 39 in the control group. The baseline characteristics of the total study population are presented in Table 3. The spherical component ranged from −11.75 to −5.50 D (mean, −7.87 ± 1.47 D) in the mRGP group, and from −11.75 to −5.25 D (mean, −7.98 ± 1.45 D) in the control group. There were no significant differences in age, sex, spherical component (non-cycloplegic refraction), or axial length between the groups at baseline (all *p* > 0.05, unpaired t- and chi-square tests).

**Table 3:** Baseline characteristics of the study population.

	Multifocal RGP group	Control group	value of p
Total number of children (eyes)	*N* = 38 (75 eyes)	*N* = 39 (75 eyes)	
Age (y)	13.47 ± 2.44	13.26 ± 2.21	0.683^*^
M/F	12/26	13/26	0.869^†^
Spherical component (D)	−7.87 ± 1.47	−7.98 ± 1.45	0.656^*^
Axial length (mm)	26.78 ± 1.21	26.75 ± 0.84	0.80^*^

Data are presented as numbers or mean ± standard deviation. *Unpaired *t*- and ^†^chi-square tests; RGP, rigid gas-permeable; D, diopter.

For the treatment zone and pupil diameter analyses, six eyes from the mRGP group were excluded because of poor-quality corneal topography; thus, 19 eyes were included in the analyses. The mean treatment zone diameter was 4.55 ± 0.25 mm (range, 4.06–4.93 mm), and the mean pupil diameter was 4.15 ± 0.46 mm (range, 3.48–5.08 mm).

The mean axial length increased from 26.78 ± 1.21 mm at baseline to 26.99 ± 1.19 mm at 1-year follow-up in the mRGP group, and from 26.74 ± 0.84 mm to 26.96 ± 0.85 mm in the control group. Hence, axial elongation was 0.20 ± 0.17 mm and 0.21 ± 0.14 mm in the mRGP and control groups, respectively, with no significant differences between the groups using univariate covariance analyses when baseline axial length was a covariate (*F* = 0.044, *p* = 0.835).

A total of 41 patients completed 2 years of follow-up: 17 in the mRGP group and 24 in the control group. Their baseline demographic and biometric data are shown in Table 4. The spherical component ranged from −11.75 to −5.75 D (mean, −8.12 ± 1.71 D) in the mRGP group and from −11.75 to −5.75 D (mean, −8.15 ± 1.55 D) in the control group. No significant differences in sex, age, spherical component, or axial length at baseline were observed between the groups (all *p* > 0.05, unpaired *t*- and chi-square tests).

**Table 4:** Baseline demographic and biometric data of patients who completed the 2-year follow-up.

	Multifocal RGP group	Control group	Value of *p*
Total	*N* = 17 (32 eyes)	*N* = 24 (48 eyes)	
Age (y)	13.41 ± 2.12	13.13 ± 1.62	0.627^*^
M/F	8/9	9/15	0.540^†^
SE (D)	−8.12 ± 1.71	−8.15 ± 1.55	0.938^*^
AL (mm)	26.97 ± 1.27	26.90 ± 1.02	0.788^*^

Data are presented as numbers or mean ± standard deviation. *Unpaired *t*- and ^†^chi-square tests. SE, spherical equivalent; AL, axial length; D, diopter.

At the 1- and 2-year follow-up visits, axial length increased by 0.21 ± 0.15 mm and 0.37 ± 0.27 mm, respectively, from the baseline value in the mRGP group, and by 0.24 ± 0.13 mm and 0.43 ± 0.23 mm, respectively, in the control group. Compared with the baseline values, the axial length significantly increased in both groups over the study period (*F* = 174.88, *p* < 0.001, repeated-measures analysis of variance). However, there were no significant differences in axial elongation between the groups during the 2-year period (*F* = 1.499, *p* = 0.224, repeated-measures analysis of variance).

Notably, none of the eyes developed infectious keratitis or conjunctivitis. In the mRGP group, corneal grade 1 staining was observed in 5 eyes of 3 patients after lens wear. However, the corneal staining improved after administration artificial tears.

## 4. Discussion

In this study, axial length growth effected by mRGP lenses with mid-peripheral zones and added high plus power, designed specifically to control myopia, was compared to that effected by single-vision spectacles in children and adolescents over a 2-year period. While axial elongation in the mRGP group was lower than that in the control group over the follow-up period, no clinically significant differences between the groups were identified.

According to the existing literature, several methods available for controlling low-to-moderate myopia include spectacles with novel technology and designs, different doses of topical atropine, overnight orthokeratology lenses, and MFSCLs (24, 26, 37). In the past few years, novel designed of spectacle lens have demonstrated significant myopia control effects on low to moderate myopic children. DIMS lenses are comprised of a central optical zone for correcting distance refractive errors, and an annular multiple focal zone with multiple segments that with a relative positive power of 3.50 diopters and 1 mm diameter. The results with a 2-year randomized controlled trial (RCT) and a third-year of non-randomized follow-up study (Children who had worn DIMS lenses in the RCT continued to wear DIMS lenses and The children in the original control group were offered the DIMS treatment in the third Year) reported that the overall control effect over a 3-year period was a reduction of myopia by 0.71D and a decrease of 0.37 mm in axial length that compared with those in the single vision group (16, 17). Furthermore, Bao et al. reported the results of a 2-year RCT, compared to the control group, which showed a significant reduction in HAL group with myopia progression by 0.80 D and axial length elongation of 0.35 mm (12). A systematic review article indicated that in terms of controlling axial elongation over a period of 2 years, high-dose atropine (≥0.5%) and moderate-dose atropine (0.1 to 0.5%) were more effective than orthokeratology lenses, but orthokeratology lenses were more effective than low-dose atropine (<0.1%), while low-dose atropine showed similar effectiveness to MFSCLs. On the other hand, peripheral plus spectacles showed slightly weaker efficacy in controlling myopia compared to orthokeratology lenses, but they were more effective than low-dose atropine (37). Several studies over the past two years have indicated that the combined use of low-dose atropine and orthokeratology lenses has a significantly greater effect on myopia control compared with orthokeratology therapy alone (38–40). Notably, these studies mostly focused on low-to-moderate myopia, and only one early study reported that moderate-concentration atropine eye drops (0.5%) were effective for controlling high myopia (≤ −6.0 D). The study included a small sample of 20 children aged 7–14 years, with baseline myopia ranging from −6.25 D to −12.00 D. Prior to atropine treatment, myopia progression was −0.14 D /M (diopter/month, SD, 0.07), and after treatment, myopia progression was −0.04 D/M (SD 0.06). However, they did not compare axial length (33). Additionally, the side effects of atropine limit its clinical application. Atropine, a non-selective antagonist of the muscarinic acetylcholine receptor, works by blocking the receptor and preventing the proliferation of scleral fibroblasts, thereby inhibits the axial elongation of the eye (41). Based on this mechanism, it is expected that atropine should also be able to control the progression of high myopia. However, the specific concentration of atropine to achieve optimal results in controlling high myopia is still a matter of concern and requires further research.

Zhou et al. reported the efficacy of wearing orthokeratology lenses for 5 years in 30 adolescents with an average age of 15. Lenses were worn two ways: day wear (greater than −6.50 D) and night wear (less than −6.50 D). Compared with before wearing orthokeratology lenses, refractive errors and axial length change after orthokeratology was not significantly different in the 5-year follow-up period (42). Based on previous studies (42, 43), the maximum degree of myopia correction achievable through fully- corrected orthokeratology was within −7.50 D. There are some concerns that overcorrection could cause corneal staining, affecting the safety and efficacy of orthokeratology. Charm et al. proposed partial reduction orthokeratology as a method of controlling high myopia and found that axial elongation in children wearing these lenses was 63% slower than that in children wearing spectacles (32). Zhu et al. further confirmed the effectiveness of this method for controlling high myopia progression (36). The disadvantage of partial reduction orthokeratology correction is that children have to wear spectacles in the daytime. Therefore, full correction of high myopia would be more achievable if a new orthokeratology lens design was commercially available. Thus, preventing the development of high-to-super-high myopia remains challenging for clinical practitioners.

The mechanisms underpinning myopia control, such as with contact lenses, are based on alterations in the retinal peripheral defocus. Several studies have reported that the eyes can respond to myopic and hyperopic defocusing via modified axial length (44–46). In addition, hyperopic defocus in the peripheral retina can drive axial length growth and myopia progression. Contemporary reverse-geometry lenses are designed to induce central corneal flattening and mid-peripheral corneal steepening, thus generating clear foveal vision while concurrently inducing myopic shifts on the peripheral retina. The mRGP lenses used in this study were similar in design to orthokeratology lenses, but the back base- curve was designed to be parallel to the corneal shape and not to flatten the central cornea, and a plus lens was added to the mid-peripheral curve on the anterior surface to induce myopic defocus shifts on the peripheral retina. For orthokeratology lenses, optical surface alterations depend not only on the lens design but also on corneal responses. For the mRGP lens, optical surface alterations are mainly determined by the lens design, which is a sharply rising edge in the mRGP lens profile. Thus, mRGP lens induces myopic defocus shifts on the peripheral retina when worn. However, the clinical impact of this type of lens remains unclear. In the present study, no clinically significant differences in axial elongation were recorded between the groups. It remains unclear why mRGP lenses cannot prevent axial growth and control myopia progression, although this finding may be partially explained by orthokeratology effects.

Several studies that quantitatively examined axial length using the defocus characteristics produced during treatment found that several factors affect the control of myopia progression when using orthokeratology lenses: such factors include baseline age (28, 30), initial spherical equivalent (30), and pupil areas (47). The treatment zone (48, 49) and high-order aberrations (50) also have important roles in slowing axial elongation. In 2016, Allinjawi et al. compared the peripheral retinal hyperopic defocus reductions with two different progressive multifocal contact lens designs and reported that lenses with additional power commencing at 3.5 mm diameter induced significant reduction in the peripheral retinal hyperopic defocus compared with lenses with additional power commencing at 5.0 mm diameter (51). A recent study demonstrated that a smaller back optic zone diameter for the orthokeratology lens generated a smaller plus power ring diameter on the anterior corneal surface. When the plus power ring horizontal sector was inside the pupils, the mean axial elongation was 76% less than when it was outside the pupils (49). Jiang et al. reported that multifocal soft contact lenses with smaller central treatment zones and closer adjacent additional power putatively improved myopia progression (52).

Furthermore, orthokeratology lenses have been reported to have greater treatment efficacy in children with large pupil diameters (47). Thus, natural pupil size may be an important factor that regulates eye growth in terms of myopia control interventions: the larger the pupil diameter, the less the axial elongation. A possible explanation for this may be that larger pupils have greater defocus volumes, and the retina may receive more peripheral myopic defocus signals induced by orthokeratology lenses as protective factors for myopia control. Combined orthokeratology and atropine therapy improved myopia control when compared with orthokeratology alone; thus, this combination treatment may increase photopic pupil size and higher-order aberrations but reduce axial growth (53).

Orthokeratology lenses flatten the central zone of the cornea and steepen the mid-peripheral zone during overnight wear. During the day, the lenses are removed, but the corneal anterior surface remains altered, and corneal refractive power shift is induced (54). The changes in corneal refractive power can be captured using corneal topography. In this study, relative plus refractive errors to the center were identified on mid-peripheral topography when patients wore contact lenses. In the mRGP group, the central zone was 5.0 mm in diameter, and the real average treatment zone size was 4.55 mm away from the corneal center, as derived from the tangential difference map, which is greater than the pupillary diameter of most patients. The mean pupil size in the 19 eyes in this study, based on the topographic maps, was 4.15 mm, although the map readings did not show good repeatability. The average photopic pupil diameter varied from 3 to 4 mm in previous studies (53, 55). According to another previous study, the average difference in pupil diameters between scotopic and photopic conditions was 1.5 mm (47). Therefore, the pupil sizes measured in this study approximate the pupil size in normal indoor conditions. It was judged that the treatment zones in mRGP lenses were greater than the pupillary diameters and induced defocus rings outside the pupils. Yang et al. showed that smaller central treatment zone diameters in topographic tangential maps exerted greater effects on axial elongation slowdown (56). Recently, a study reported that orthokeratology lenses designed with a smaller back optic zone diameter induced a reduced plus power ring diameter and improved axial elongation slowdown when compared with standard orthokeratology lenses (49). Therefore, myopia control using multifocal contact lenses may be related to the lens design, mainly to the base curve diameters. It is possible that mRGP lenses designed with a 4.0 mm central zone could slow down axial elongation more than those designed with a 5.0 mm central zone.

In addition to the aforementioned factors, the additional lens power must be considered. In the current study, the additional power in the front mid-peripheral lens was +9 D, and the amount of power added was not varied with the central refractive power. It is unclear whether the added power produces sufficient retinal myopic defocus to inhibit axial elongation in high myopia. A 2-year randomized study showed that the baseline spherical equivalent in orthokeratology was significantly associated with axial length growth (30); thus, children with high myopia benefited from orthokeratology lenses more than children with low myopia. One hypothesis explaining this effect is that eyes with high myopia have a greater degree of corneal steepening in the mid-periphery, which has a beneficial effect on the peripheral retinal defocus, thereby slowing axial length growth and myopia progression. A 3-year multicenter double-blind randomized controlled trial conducted by Walline et al. found that high-addition (+2.50 D) multifocal soft contact lenses, compared to medium-addition (+1.50 D) multifocal soft contact lenses, delayed myopia progression by 0.3 D and slowed axial elongation by 0.16 mm. The high-addition multifocal soft contact lenses significantly reduced the rate of myopia progression over a 3-year period (14). Another meta-analysis reached a similar conclusion: when the addition power increased to +2.50 D, multifocal soft contact lenses may have a significant improvement in myopia control effectiveness (57). In animal studies, the dose-dependent effects of optical interventions in limiting lens-induced myopia were mainly attributed to lens design characteristics, including addition and area of lens addition (58), peripheral defocus, and lens asphericity (59). Thus, mRGP lens treatment may increase the added power due to peripheral refraction, although this may be due to retinal profiles. However, if too many plus lenses are added to the mid-periphery, the visual quality may decrease, leading to blurring of vision, ghost images, dizziness, and headaches.

In addition to the possibility of inadequate retinal myopic defocus induced by the lens design of mRGP, other factors that could potentially affect study finding were the initial age and spherical equivalent values of children when they started wearing mRGP lenses.

The limitations of this study are as follows. First, this study included a short follow-up period and a small sample size, with even fewer patients included in the second year. Based on the research findings, it can be observed that the difference in mean axial elongation between the two groups was greater in the second year than in the first year (0.06 mm vs. 0.03 mm). If both groups had an adequate sample size and a longer follow-up period, it could potentially lead to different outcomes. Second, changes in the relative peripheral refraction in patients wearing mRGP lenses were not measured. Perhaps the induced peripheral retinal myopic defocus was insufficient to inhibit myopia progression for highly myopic eyes with refractive errors as high as −10.00 D. However, selected patients wearing mRGP lenses had high and super-high myopia, and although their average age was 13 years, they had not passed their peak progression. For example, axial length growth in the control group was 0.24 mm and 0.43 mm at the 1- and 2-year visits, respectively. Thus, the pathogenesis of myopia in these patients may differ from that in patients with low-to-moderate myopia. Finally, this study was retrospective in nature, which may have introduced selection bias. For instance, the choice of corrective method was not randomly assigned to the two groups of patients; patients in the mRGP group requested mRGP lenses for vision correction, which is most likely due to the fact that they had a faster myopia progression compared to patients in the spectacle group before wearing RGP lenses, and their myopia was more prone to further progression. These factors could have impacted the result of our study. Therefore, future prospective studies with larger sample sizes and longer follow-up periods should be conducted to determine whether mRGP lenses can slow myopia progression in younger children.

## 5. Conclusion

Contemporary evidence suggests that mRGP lenses have no significant impact on controlling myopia progression compared with spectacles. Based on our findings, lens manufacturers may need to alter their designs to increase the amount of retinal myopic focus. Manufacturers can gain valuable design insights from MFSCLs or orthokeratology lens to optimize the design of mRGP lens. It has been suggested that MFSCLs can manipulate a broader range of optical defocus, leading to a greater degree of myopia control (14, 57). In this study, mRGP lenses employed a progressive lens design (peripheral add lens design), which involves a gradual change in the curvature of the anterior surface of the lens. This design provides a central zone for distance correction, while the progressive change includes a relatively positive power in the periphery. Improvements in mRGP lens design can be achieved by appropriately increasing the positive add power of the lens. However, it is important to avoid excessive plus power that could significantly compromise visual quality. Additionally, considering an aspherical design for the central zone of the lens or reducing the base curve diameter to 3–4 mm might be beneficial. Furthermore, enhancing the design to widen and steepen the reverse curve zone can allow for a greater volume of defocus to reach the retina. Alternatively, another design option is to utilize the concentric ring design or bifocal design of MFSCLs. Currently, practitioners have limited measures in controlling the progression of high myopia. It is hoped that manufacturers can improve the design of mRGP lenses, and a rigorous prospective comparative study will be conducted by our research team. Specifically, this study will include individual with myopia ranging from −6.00 D to −10.00 D, within a narrower age range, such as 7–13 years old, with an adequate sample size. Genetic factors would be excluded to effectively evaluate the actual effectiveness of mRGP lenses in controlling high myopia.

## Data availability statement

The raw data supporting the conclusions of this article will be made available by the authors, without undue reservation.

## Ethics statement

The studies involving humans were approved by the ethics committee of Eye Hospital of Wenzhou Medical University. The studies were conducted in accordance with the local legislation and institutional requirements. Written informed consent for participation in this study was provided by the participants’ legal guardians/next of kin.

## Author contributions

L-hY and W-qJ: study design and manuscript writing. RZ, G-xS, and ML: data collection and analysis. All authors contributed to manuscript revision and read and approved the submitted version.

## Funding

This study was supported by Innovation and Guidance project of Eye Hospital of Wenzhou Medical University (YNCX3201907).

## Conflict of interest

The authors declare that the research was conducted in the absence of any commercial or financial relationships that could be construed as a potential conflict of interest.

## Publisher’s note

All claims expressed in this article are solely those of the authors and do not necessarily represent those of their affiliated organizations, or those of the publisher, the editors and the reviewers. Any product that may be evaluated in this article, or claim that may be made by its manufacturer, is not guaranteed or endorsed by the publisher.

## Abbreviations

mRGP: Multifocal rigid gas-permeable
RGP: Rigid gas-permeable
